# Commentary: We Need to Change: Integrating Psychological Perspectives Into the Multilevel Perspective on Socio-Ecological Transformations

**DOI:** 10.3389/fpsyg.2021.724768

**Published:** 2021-08-18

**Authors:** Daniel Hanss

**Affiliations:** Department of Social Sciences, Darmstadt University of Applied Sciences, Darmstadt, Germany

**Keywords:** transformation research, socio-technical transition, multilevel perspective, agency, environmental psychology

## Introduction

In their opinion article, Wullenkord and Hamann (2021) propose research avenues for increasing psychology's relevance for and impact in transformation research. One of their suggestions is to integrate constructs of psychological agency theories into an extended version (Göpel, 2016) of the multi-level perspective (MLP; Geels and Schot, 2007). My opinion is that this approach can give psychology some general guidance, for example, as to whether existing agency research sufficiently considers different structural levels that characterize socio-technical transitions. One likely insight will be that psychology needs to pay more attention to group-level constructs and concepts at the intersection of psychology and sociology [as suggested by Upham et al. (2020); Ruhrort and Allert (2021), albeit unrelated to MLP]. Efforts to explore the role of social (Schulte et al., 2020) or global identity (Loy et al., 2021) for individual and collective pro-environmental behaviors are examples of steps toward closing this research gap. However, I also think that the proposed theory integration has limitations that deserve mentioning:

- One limitation concerns the advancement of MLP through auxiliary theories. While Geels (2011) points out the potential value of including insights on agency into MLP, he also stresses that open, heuristic frameworks are better suited for studying multi-dimensional topics—like socio-technical transitions—than rigorous, mathematical explanatory models. Psychological agency theories that link behavior causally to specific underling factors may, thus, be incompatible with MLP.
- Another limitation concerns the scope of agency perspectives for application in transformation research. Wullenkord and Hamann (2021) emphasize that psychology needs to pay more attention to processes and events in system transformations to increase its practical value for transformation research. I agree but think that their approach to contemplate psychological agency perspectives within a general explanatory framework like MLP risks ignoring system characteristics essential to understanding agency in transformations, many of which likely depend on the domain and context in which a transformation takes place. Ignoring these characteristics may lead to premature conclusions about the relevance of psychological constructs.

More comprehensive insights for psychological contributions can evolve from analyses of specific systems and transformations that consider the contextual embeddedness of actors and behaviors. These analyses are essential elements of inter- and transdisciplinary transformation research initiatives and may be guided by MLP. For psychology to become more transformation-oriented, psychologists need to actively engage in these initiatives and help advance solutions toward their common practices and challenges.

Below, I will give a brief overview of different strands of transformation research. I will then provide examples of how psychology can support these research strands through agency-related concepts and research. I, thereby, hope to complement Wullenkord and Hamann's 2021 and other (e.g., Upham et al., 2020; Bruhn, 2021) recent contributions on the relevance of psychology for transformation research.

## Strands of Transformation Research

Transformation research can be broadly distinguished by the *mode* in which research is conducted: *problem-oriented, descriptive-analytical* vs. *solution-oriented, transformative* (Wittmayer and Hölscher, 2017). The former mode investigates the complexity and dynamics of systems (e.g., socio-ecological or socio-technical) underlying sustainability-related challenges, by integrating perspectives of different scientific disciplines. The latter mode builds upon and goes beyond problem description and analysis. It strives to develop, test, and implement practical solutions to sustainability-related challenges in collaborative fashion by integrating insights from different scientific disciplines and expertise of societal actors (Wiek et al., 2012). During this process, solution options are evaluated on impact indicators, like carbon emission estimates from life cycle analyses, and only pursued, if they promise significant improvements in the targeted system. Another way to distinguish transformation research is with regard to the *system under investigation*. For example, sustainability science focusses on *socio-ecological* and transition research on *socio-technical* or *socio-economic* systems (Wittmayer and Hölscher, 2017), with each line of research using different analytic frameworks.

One such framework, rooted in transition research, is MLP. Geels (2011) refers to MLP as a “heuristic device” to help analysts derive conclusions about events and dynamic patterns in transitions by pointing them to relevant questions and problems about the system under investigation. Among these questions and problems are such relating to the identification of transition-relevant actors, behaviors, and their influencing factors.

## How Transformation Research Initiatives Can Benefit From Psychological Agency Concepts

Problem-oriented, descriptive analytical initiatives may ask what contextual and psychological factors underlie agency in an “unsustainable” socio-technological system, such as electricity or transport in a confined geographical region. These systems and their transitions are influenced by multiple individual and group actors (e.g., consumers, policymakers, companies), with potentially distinct constellations of interests, beliefs, or strategies, and involve various types of agency (Köhler et al., 2019), likely unique to specific systems. Furthermore, transformation-relevant behaviors are embedded in institutions (i.e., formal and informal rules), spatial arrangements (e.g., infrastructure, urban design characteristics), and cultural contexts (Di Giulio et al., 2014). For example, people's choice of transportation may be affected by formal parking space regulations in their neighborhoods or at their workplaces, while informal rules could, e.g., develop from conversations about mobility and livable urban spaces taking place in local citizen networks. Transport decisions may also depend on how much public space is attributed to different transport modes or how residential, commercial, and recreational areas are spatially organized in communities and the resulting distances that people need to travel in everyday life. While spatial organization of public spaces may be culture specific, cultural influences could, e.g., also stem from status connotations of different transport modes. To shed light on these various aspects, analyses of the system at hand are warranted before psychological constructs can be meaningfully selected for the study of agency. System analyses can be guided by MLP (see, e.g., Nykvist and Whitmarsh, 2008) and should provide sufficient detail on the contextual embeddedness of the relevant actors and behaviors, to inform assumptions about which psychological constructs need be considered. If contextual and psychological factors are integrated into explanatory behavior models and put to empirical test, insights from these studies can inform more comprehensive descriptions of the respective system and prospects of how it may be transformed.

Solution-oriented, transformative initiatives may strive to facilitate niche innovations in a concrete socio-technical system and involve analyses (e.g., guided by MLP) of the context and actors relevant to collaborative development and implementation of the innovations. Collaboration can be conducted at varying degrees of distance, with more proximate approaches building upon bidirectional consulting and learning between researchers and societal actors (Lang and Wiek, 2021). Researchers in such transdisciplinary initiatives will face challenges like actively engaging societal actors with relevant expertise and influence—but different roles (e.g., representatives of companies, municipal administration, citizens' initiatives), professional backgrounds, and motivations—for collaboration in niches. In later transition stages, researchers may need to build ownership and intent among implementers and potential adopters of the niche innovations. Psychological agency concepts and research can inform these efforts, for example, with insights on how to strengthen individual and group-level determinants of niche-actor engagement (Hamann et al., 2021) or adoption of niche-innovation among users in the regime (Keller et al., 2021). Such psychological contributions can draw from and feed back into descriptive-analytical transformation research.

## Discussion

To increase its relevance for and impact in transformation research, psychology needs to embrace the complexity and context embeddedness of agency in system transitions. Another recent initiative concerned with impact orientation in environmental psychology has stressed that contextual factors are particularly important for explaining high-impact behaviors, probably more so than the attitude(like) constructs covered by prevailing psychological agency theories (Lange et al., 2021; Nielsen et al., 2021). The authors, consequently, argue for explanatory approaches to studying high impact behaviors and inductive development of agency theories.

My suggestions for investigating agency in system transformations share commonalities with this approach. In summary, I recommend that psychologists build upon system analyses when they explore which factors affect relevant actors and behaviors. This will facilitate the development of explanatory models for high impact behaviors—from a transformation research viewpoint—that integrate contextual and psychological factors. Initially, these models will be geared toward specific cases (i.e., actors, behaviors, systems, transformations). Through comparisons across specific cases, more general insights on agency in system transformations could evolve that may be better suited for integration into heuristic frameworks, like MLP, than current psychological agency theories.

## Author Contributions

The author confirms being the sole contributor of this work and has approved it for publication.

## Conflict of Interest

The author declares that the research was conducted in the absence of any commercial or financial relationships that could be construed as a potential conflict of interest.

## Publisher's Note

All claims expressed in this article are solely those of the authors and do not necessarily represent those of their affiliated organizations, or those of the publisher, the editors and the reviewers. Any product that may be evaluated in this article, or claim that may be made by its manufacturer, is not guaranteed or endorsed by the publisher.
